# Poor People Are Hospitalized Three Times More for Mental Health Services than the Non-Poor in Central Valley California

**DOI:** 10.3390/healthcare6010005

**Published:** 2018-01-12

**Authors:** Gyanesh Lama, Emanuel Alcala, John A. Capitman

**Affiliations:** 1 Department of Social Work Education, California State University, 5310 N. Campus Drive, M/S PHS 102, Fresno, CA 93710-8019, USA; 2 Central Valley Health Policy Institute, California State University, Fresno, CA 93710-8019, USA; ealcala@csufresno.edu (E.A.); jcapitman@csufresno.edu (J.A.C.)

**Keywords:** poverty, health insurance, mental health, hospitalization

## Abstract

**Introduction:** Providing health insurance to the poor has become a standard policy response to health disparities between the poor and the non-poor. It is often assumed that if the poor people are given health insurance, they will use preventative care, which will prevent more expensive emergency visits and inpatient hospitalization, and in turn, it will save healthcare cost in the long run. This paper presents the findings from our study in California about what happens to the poor when they are given health insurance. The purpose of the study was to understand how the healthcare system in California treats the poor patients differently than the non-poor. **Method:** Using multivariate logistic regressions, this study analyzed a large patient discharge data (PDD) from the California Office of Statewide Planning and Development (OSHPD) for eight counties in the Central Valley California (N = 423,640). First, utilizing International Classification of Diseases (ICD 10) as diagnostic criteria, mental-health vs. non-mental health hospitalization rates were estimated. Second, health insurance status was used as a proxy measure of poverty of the patients. Using chi-Square, the probability of hospitalization for mental health services was estimated based on their insurance types. Finally, using step-wise logistic regression, the odds of mental health hospitalization was estimated conditional on individual characteristics, health insurance types, and geographic characteristics. **Findings:** When the poor people were given health insurance, they were three times more likely to be hospitalized for mental health services than the non-poor. The more than three-fold variation in mental health hospitalization was not driven by demographic or geographic characteristics. The findings are new and have important implications for the healthcare policies for the poor. Further studies are needed to understand the extent to which the disproportionately high rate of mental health hospitalizations of the poor are driven by the provider-induced needs.

## 1. Introduction

The Central Valley California has the highest concentration of poverty in the U.S. [1,2]. It also has high disease rates and health disparities [3]. Poverty has serious effects on the health of the poor [4], and the poor people in Central Valley California suffer from a disproportionately high level of diseases burdens and low quality of life. The Central Valley California, therefore, presents itself as a perfect natural laboratory to study how poor people are being treated by the current healthcare system.

It is often believed that poor people have higher level of health problems because they lack access to healthcare services. Studies show, for example, that the poor are less likely to receive mental health services than the non-poor and face barriers when trying to access care [5,6]. The primary reason is that the poor people do not have health insurance [7]. 

Providing health insurance to the poor, therefore, has become a policy priority of the government as a response to the disparities in healthcare between the poor and the non-poor. The often-used logic is that, if poor people are given health insurance, they will use preventative care, which will prevent more expensive emergency visits and inpatient hospitalization. As a result, it is assumed, it will save more money for the government in the long run (e.g., [8,9], etc.). 

Health insurance, therefore, has become a marker of access to healthcare services. While much has been written about the under-utilization of health services by the poor due to the lack of insurance, very little is known about what actually happens when the poor people are given health insurance. Do they utilize health services as predicted? Does it reduce inpatient hospitalization? Using health insurance type as a proxy measure of poverty status of the patients, this study examined the pattern of inpatient hospitalization for health services among the poor in Central Valley California. The study analyzed hospital data (N = 423,640) from the California Office of Statewide Planning and Development (OSHPD). The purpose of the study was to understand how the healthcare system in California treats poor patients in comparison to non-poor patients. 

## 2. Methods

This was a retrospective observational study composed of all patient discharge data (PDD) from the California Office of Statewide Planning and Development (OSHPD) data set. Hospitals in California are mandated to report all patient discharges to OSHPD. These reports include the patient’s age, race/ethnicity, sex, county and zip code of residence, expected source of payment, hospital charges, facility type, up to 24 diagnoses, and 24 procedure codes. The most recent data set available was from 2012, as data are de-identified and made publicly available one to two years after a patient has been discharged. These data were obtained with approval from the Committee for the Protection of Human Subjects (CPHS) of California’s Health and Human Services Agency. The current study evaluated records of those residing within the eight San Joaquin Valley (SJV) counties: Fresno, Kern, Kings, Madera, Merced, San Joaquin, Stanislaus, and Tulare. 

### 2.1. Measures 

The Clinical Classifications Software (CCS) for ICD-9CM was used to identify major diagnostic categories within the hospitalization records. This tool was developed by the Healthcare Cost and Utilization Project (HCUP), a Federal-State-Industry sponsored by the Agency for Healthcare Research and Quality (AHRQ). A patient’s principal diagnosis was attributed to the first-listed International Classification of Diseases, Ninth Revision, Clinical Modification (ICD-9-CM) code. Only the principal diagnosis, ICD-9-CM codes 290-319, was used to identify hospitalizations due to a mental disorder. Patient records are de-identified; therefore, it was not possible to evaluate readmissions in any capacity. All hospitalizations were recoded into one of two categories (0 = Any Other Condition and 1 = Mental Disorder).

The patient discharge data (PDD) included information on insurance status (payer category) of the patients and classified it into four categories: (1) *Private* (includes self-pay and workers’ compensation); (2) *Medicare*; (3) *Medi-Cal*, and (4) *Government* (includes other government, indigent, and county indigent programs provided for the poor). In this study, we used the *Government* insurance as a proxy measure of poverty status of the patients. Although *Medi-Cal* is largely for the poor, in California, it also covers those who are aged and disabled. Therefore, we treated *Medi-Cal* as a separate category from the *Government*, which was specifically designated for the poor. This allowed us to be more precise in our comparison across insurance types. 

Characteristics of the individual were dummy-coded and included gender, age group, and race/ethnicity. For the variable gender, male was used as the reference group (0 = Male and 1 = Female). Age was categorized into three levels: 20–64, 65 and older, and under 20 as the reference group. Racial/ethnic groups included Black, Hispanic, Asian, Other, and White as the reference group. The patient’s county of residence was included to examine regional differences in mental services. The eight counties included compose California’s SJV and the reference group, Madera, was chosen due to mean rates of mental disorder hospitalizations. 

### 2.2. Data Analysis

Bivariate analysis was used to examine initial associations between mental disorder hospitalizations and the type of insurance. Insurance types were collapsed to reflect both proportions of use and insurance characteristics. Collapsing the insurance types into broader, more inclusive, groups did not significantly impact subsequent analyses. Based on the bivariate results, we then constructed models testing associations between mental disorder hospitalizations and independent variables using a logistic regression.

We used a direct model building strategy to produce three analytic logistic regression models. The first model included only types of insurance. The second model included gender, age, and race/ethnicity covariates, in addition to the types of insurance. Lastly, the final model introduced the patient’s county of residence in an attempt to estimate geographical variation. Strong multicollinearity was not found, but all other model assumptions were met. Model over-fitting was not an issue due to our large sample size and maintained an event-to-covariate ratio greater than 20:1. Data analysis was performed using IBM SPSS Statistics for Windows, Version 22.0. (IBM Corp., Armonk, NY, USA).

## 3. Results

Table 1 presents the data on characteristics of the study population. Among all the hospitalized population, 19,178 (4.5%) were hospitalized for mental health, 59.3% were female, and race/ethnicity variables were as follows: 46.2% White, 40% Hispanic, 6.1% Black, 4.8% Asian, and 3% Other. The majority (49.6%) were 20–64 years old, followed by 65 and over (25.8%) and 24.6% under 20. Majority (35.8%) of the patients had Medi-Cal, 30.4% had private insurance, 29.3% had Medicare, and 4.6% had government insurance. Most of the patients were residents of Fresno County (24.5%), followed by Kern (21.4%), San Joaquin (16.1%), Stanislaus (13.8%), Tulare (11.3%), Merced (6.0%), Madera (3.5%), and Kings (3.4%). 

Table 2 presents the data on reasons for hospitalization by insurance type. The data show that, among those with government insurance (poor), 15.3% were hospitalized for mental health, compared to only 4.7% among those with private insurance (non-poor), 4.5% among Medi-Cal, and 2.7% among Medicare users. 

Table 3 presents the results of the multivariate logistic regression. The results of the multivariate logistic regression show that the odds of mental health hospitalization among those with government insurance (poor) was 3.6 times higher than those with private insurance (non-poor) (OR = 3.63, *p* < 0.005). In other words, the odds that a patient will be hospitalized for mental health was 260% higher if he had a government insurance (poor) compared to if he had a private insurance (non-poor). In contrast, the odds of mental health hospitalization was lower for Medicare (OR = 0.56, *p* < 0.001) and Medi-Cal (OR = 0.94, *p* < 0.005) users. 

The differences continued even after controlling for demographic and geographic variables. Gender, age, race, and county of residence were significant predictors of mental health hospitalization. Women were less likely to be hospitalized for mental health than men (OR = 0.569, *p* < 0.001), the 20–64 age group were more likely (OR = 2.746, *p* < 0.001), but the over 64 age group were less likely (OR = 0.293, *p* < 0.001) to be hospitalized for mental health than those younger than 20 years old. There was no difference between White and Black in the odds of being hospitalized for mental health (OR = 1.043, *p* > 0.05); however, the odds of hospitalization for mental health was less for Hispanic (OR = 0.510, *p* < 0.001), Asian (OR = 0.559, *p* < 0.001), and Other (OR = 0.875, *p* < 0.001). County of residence was also a significant predictor of mental health hospitalization. The more than threefold differences in mental health hospitalization between different insurance types were not solely driven by the differences in patient’s demographic characteristics or geographic variables. 

## 4. Discussion 

This is the first study reporting on how the healthcare system treats poor patients differently than the non-poor in the Central Valley California. The findings from this study show that people with government insurance (the poor) were three times more likely to be hospitalized for mental health services than those with the private or other types of insurance (the non-poor). The more than threefold differences in mental health hospitalization was not driven by the differences in patient’s demographic characteristics (gender, age, and race) or geographic variables. We consider these findings new and significant, with important implications for the current healthcare system and practices. The findings also suggest that being female, having an older age, being Hispanic, Asian, or Other, and residing in Kings or Tulare counties all decrease the odds of hospitalization for mental health services. On the other hand, being 20–64 and living in Fresno or Stanislaus counties increase the odds of hospitalization for mental health services. Furthermore, this study reveals that the patient characteristics listed above seem to be evaluated differently for poor patients (those with government insurance) compared to non-poor patients (those with private insurance), thereby increasing our understanding of exactly why these variations occur. 

Our findings suggest that providing health insurance to the poor does not reduce in-patient hospitalization. In fact, when the poor were given health insurance, they were over three times more likely to be hospitalized. Controlling for demographic characteristics and county of residence, the poor (those with government insurance) were 3.2 times, those with Medicare 1.4 times, and those with Medi-Cal 1.3 times more likely to be hospitalized for mental health services than the non-poor (those with private health insurance). One explanation for this is that the poor must have more mental health problems requiring them more hospitalization than the non-poor. Poor people often face many life difficulties that cause them mental health problems (e.g. [10,11,12,13,14]), and the types and the severity of mental health problems faced by the poor may be different from those faced by the non-poor [13]. The fact that the patients from poor counties (Fresno and Stanislaus) were at higher risk of being hospitalized for mental health services than those from the non-poor counties provides further support for this explanation. 

Another plausible explanation is that the healthcare system offers different health services to its patients based on their health insurance types. Since there is no reason to believe why someone with the government insurance should have more mental problems than those with private insurance, the reason for differential treatment across insurance types must be something other than the differences in the disease conditions. One such possible reason is the differences in reimbursement rates across insurance types. Because the reimbursement rate from the government insurance is known to be lower than the private insurance, it might be the case that health providers sell the poor more services than necessary so as to make profits? From this study, it is not clear the extent to which a higher rate of hospitalization of the poor is driven by actual needs versus provider-induced needs.

The findings from this study raise more questions about the current healthcare system and practices than offer explanations. Although we have attempted to offer some explanations, the answers to the questions about why poor patients, as indicated by their insurance type, are treated differently than the non-poor are beyond the scope of this study. 

## 5. Conclusions

One implication of the findings is that providing health insurance to the poor may not reduce hospitalization and the healthcare cost. The findings provide some insights for the current thinking around health policy debate for the poor. The primary focus of the health policy debate has been one of providing health insurance to the poor as a way to reduce costs on emergency visits and to bridge health disparity (e.g., [8,9], etc.). The findings from this study show, however, that, while providing health insurance may increase access to health services, it does not appear to reduce healthcare costs. In fact, it shows the opposite effect—the poor overutilize the services increasing healthcare costs. Another implication of the findings is that the healthcare practices and system must also be considered in health policy debate. Since the healthcare system treats poor people differently than the non-poor, providing health insurance alone does not appear to be an adequate response to the needs of the poor. If the reduction in hospitalization and healthcare costs is the primary concern of the government, the root cause of the health problems—poverty—must also be addressed. 

If poverty is causing the poor to be hospitalized more, then helping the poor become non-poor seems to be the most logical and sustainable way to reduce healthcare cost in the long run. If, however, the primary interest in providing health insurance to the poor is to only increase their access to hospitals and the quality of care, then health policy debate should also consider the ways in which the health system treats the patients based on their health insurance types. 

## 6. Limitation 

The conclusions are tempered by some of the limitations of our study. First, this study used only the principal diagnosis, ICD-9-CM codes 290-319, to identify hospitalizations due to a mental disorder. Future studies should look into more detailed diagnoses by insurance type. Second, the data in this study included only the hospitals in Central Valley California. Future studies should analyze nationally representative samples, and examine the generalizability of the findings to other territories of the U.S. and other countries. Third, the OSHPD data set was de-identified and therefore did not allow the researchers to determine if there were multiple entries from the same patient. Although the chances of the multiple entries were slim according to the OSHPD expert (the co-author), to the greatest extent possible, future studies should consider analyzing the identified data. Fourth, this study used cross-sectional data analyses. Future studies should look into data for a longer period of time, which would allow for a longitudinal analysis of pattern of hospitalization of the poor over time. 

Despite these limitations, this study represents the first ever analyses of a large hospital data from the nation’s highest poverty areas in the U.S., and it is the first study to report that the poor are hospitalized three times more often for mental health services compared to the non-poor. We consider that these findings are new and significant and that they raise questions about the current healthcare system and practices. These findings underscore the need for comprehensive approaches to healthcare of the poor, ones which consider the root causes of health disparities—poverty. Such approaches are necessary to promote the health and wellbeing of all people, including the poor, and to reduce the current health disparities. Furthermore, the findings from this study show that insurance type is an important marker of the pattern of health service utilization and that any health policy debate must move beyond simply providing health insurance. The findings provide important insights about the ways healthcare systems treat patients based on economic/insurance status. 

## Figures and Tables

**Table 1:** Characteristics of hospitalized population.

Variable	N	%
**Dependent Variable**		
Reason for hospitalization		
Non-Mental Health	404,462	95.5
Mental Health	19,178	4.5
**Independent Variables**		
Gender		
Female	251,263	59.3
Male	172,353	40.7
Race/Ethnicity		
White	195,652	46.2
Hispanic	169,392	40.0
Black	26,004	6.1
Asian	20,235	4.8
Other	12,357	3.0
Age Group		
Under 20	104,352	24.6
20 to 64	210,183	49.6
65 and Over	109,105	25.8
Insurance		
Medi-Cal	151,520	35.8
Private (non-poor)	128,595	30.4
Medicare	124,016	29.3
Government (poor)	19,509	4.6
County of Residence		
Fresno	103,904	24.5
Kern	90,825	21.4
San Joaquin	68,094	16.1
Stanislaus	58,476	13.8
Tulare	47,844	11.3
Merced	25,306	6.0
Madera	14,729	3.5
Kings	14,462	3.4
Total	423,640	100.0

**Table 2:** Reasons for hospitalization by insurance type.

Insurance Type	Non-Mental Health		Mental Health		Total	
	n	%	n	%	N	%
Government (poor)	16,531	84.7%	2978	15.3%	19,509	100.0%
Private (non-poor)	122,513	95.3%	6082	4.7%	128,595	100.0%
Medi-Cal	144,756	95.5%	6764	4.5%	151,520	100.0%
Medicare	120,662	97.3%	3354	2.7%	124,016	100.0%
Total	404,462	95.5%	19,178	4.5%	423,640	100.0%

**Table 3:** Odds ratios (ORs) of mental health hospitalizations.

Parameter	Model 1		Model 2		Model 3	
	OR	95% Wald CI	OR	95% Wald CI	OR	95% Wald CI
**Insurance**						
Private (non-poor)	Reference		Reference		Reference	
Government (poor)	3.628 ***	(3.463, 3.802)	3.254 ***	(3.1, 3.414)	3.196 ***	(3.044, 3.356)
Medicare	0.559 ***	(0.536, 0.584)	1.375 ***	(1.309, 1.445)	1.399 ***	(1.331, 1.47)
Medi-Cal	0.941 **	(0.908, 0.975)	1.325 ***	(1.277, 1.376)	1.317 ***	(1.268, 1.367)
**Gender**						
Male	-	-	Reference		Reference	
Female	-	-	0.569 ***	(0.552, 0.587)	0.569 ***	(0.551, 0.586)
**Age Group**						
Under 20	-	-	Reference		Reference	
20 to 64	-	-	2.773 ***	(2.655, 2.896)	2.746 ***	(2.628, 2.868)
65 and Over	-	-	0.302 ***	(0.278, 0.328)	0.293 ***	(0.27, 0.318)
**Race/Ethnicity**						
White	-	-	Reference		Reference	
Black	-	-	0.987	(0.936, 1.041)	1.043	(0.988, 1.1)
Hispanic	-	-	0.484 ***	(0.467, 0.501)	0.510 ***	(0.492, 0.528)
Asian	-	-	0.550 ***	(0.507, 0.598)	0.559 ***	(0.514, 0.608)
Other	-	-	0.885 **	(0.814, 0.962)	0.875 ***	(0.804, 0.952)
**County of Residence**						
Madera	-	-	-	-	Reference	
Fresno	-	-	-	-	1.387 ***	(1.262, 1.524)
Kern	-	-	-	-	1.004	(0.912, 1.105)
Kings	-	-	-	-	0.582 ***	(0.508, 0.666)
Merced	-	-	-	-	0.918	(0.819, 1.030)
San Joaquin	-	-	-	-	1.061	(0.961, 1.170)
Stanislaus	-	-	-	-	2.166 ***	(3.348, 4.137)
Tulare	-	-	-	-	0.857 ***	(1.313, 1.651)

** *p* ≤ 0.005. *** *p* ≤ 0.001. Odds ratio is indicated by OR and Wald method was used to calculate 95% confidence interval and is indicated by 95% Wald CI.

## Availability of Data and Material

The data that support the findings of this study are available from California Office of Statewide Planning and Development (OSHPD), but restrictions apply to the availability of these data, which were used under license for the current study and so are not publicly available. Data are, however, available from the authors upon request and with permission from the California Office of Statewide Planning and Development (OSHPD).
